# Supplementary material for: Iterative improvement in the automatic modular design of robot swarms
Source: PeerJ Comput Sci. 2020 Dec 7;6:e322. doi: 10.7717/peerj-cs.322 (PMC7924708; doi:10.7717/peerj-cs.322)
Supplement: Supplemental Information 3 [file peerj-cs-06-322-s003.zip › argos3/doc/api/standalone/a00655_source.html]

ARGoS: plugins/simulator/visualizations/qt-opengl/qtopengl\_main\_window.h Source File


- Main Page
- Related Pages
- Namespaces
- Classes
- Files

- File List
- File Members

# plugins/simulator/visualizations/qt-opengl/qtopengl\_main\_window.h

Go to the documentation of this file.

```
00001 
00007 #ifndef QTOPENGL_MAIN_WINDOW_H
00008 #define QTOPENGL_MAIN_WINDOW_H
00009 
00010 namespace argos {
00011    class CQTOpenGLMainWindow;
00012    class CQTOpenGLWidget;
00013    class CQTOpenGLLogStream;
00014    class CQTOpenGLUserFunctions;
00015 }
00016 
00017 class QMainWindow;
00018 class QWidget;
00019 class QHBoxLayout;
00020 class QPushButton;
00021 class QLCDNumber;
00022 class QStatusBar;
00023 class QDockWidget;
00024 class QTextEdit;
00025 class QButtonGroup;
00026 class QSpinBox;
00027 class QDoubleSpinBox;
00028 class QActionGroup;
00029 
00030 #include <argos3/core/utility/configuration/argos_configuration.h>
00031 #include <argos3/core/simulator/visualization/visualization.h>
00032 #include <QMainWindow>
00033 
00034 namespace argos {
00035 
00036    class CQTOpenGLMainWindow : public QMainWindow {
00037 
00038       Q_OBJECT
00039 
00040    public:
00041 
00042       CQTOpenGLMainWindow(TConfigurationNode& t_tree);
00043       virtual ~CQTOpenGLMainWindow();
00044 
00045       inline CQTOpenGLWidget& GetOpenGLWidget() {
00046          return *m_pcOpenGLWidget;
00047       }
00048 
00049       inline const CQTOpenGLWidget& GetOpenGLWidget() const {
00050          return *m_pcOpenGLWidget;
00051       }
00052 
00053       inline CQTOpenGLUserFunctions& GetUserFunctions() {
00054          return *m_pcUserFunctions;
00055       }
00056 
00057       inline const CQTOpenGLUserFunctions& GetUserFunctions() const {
00058          return *m_pcUserFunctions;
00059       }
00060 
00061       inline const QString& GetIconDir() const {
00062          return m_strIconDir;
00063       }
00064 
00065       inline const QString& GetTextureDir() const {
00066          return m_strTextureDir;
00067       }
00068 
00069    private:
00070 
00071       void ReadSettingsPreCreation();
00072       void ReadSettingsPostCreation();
00073       void WriteSettings();
00074 
00075       void CreateExperimentActions();
00076       void CreateCameraActions();
00077       void CreatePOVRayActions();
00078       void CreateHelpActions();
00079 
00080       void CreateExperimentToolBar();
00081       void CreateExperimentMenu();
00082       void CreateCameraToolBar();
00083       void CreateCameraMenu();
00084       void CreatePOVRayMenu();
00085       void CreateHelpMenu();
00086 
00087       void CreateOpenGLWidget(TConfigurationNode& t_tree);
00088       void CreateLogMessageDock();
00089       void CreateConnections();
00090 
00091       void CreateUserFunctions(TConfigurationNode& t_tree);
00092 
00093       virtual void closeEvent(QCloseEvent* pc_event);
00094 
00095    signals:
00096 
00101       void CameraSwitched(int n_camera);
00102 
00106       void ExperimentStarted();
00107 
00111       void ExperimentPlaying();
00112 
00116       void ExperimentFastForwarding();
00117 
00125       void ExperimentPaused();
00126 
00134       void ExperimentSuspended();
00135 
00140       void ExperimentResumed();
00141 
00147       void ExperimentDone();
00148 
00152       void ExperimentReset();
00153 
00154    public slots:
00155 
00161       void PlayExperiment();
00162 
00167       void FastForwardExperiment();
00168 
00172       void StepExperiment();
00173 
00179       void PauseExperiment();
00180 
00184       void TerminateExperiment();
00185 
00190       void ResetExperiment();
00191 
00195       void SuspendExperiment();
00196 
00200       void ResumeExperiment();
00201 
00202       void CameraXMLPopUp();
00203 
00204       QString GetCameraXMLData();
00205 
00206       void SwitchCamera(QAction*);
00207 
00208       // void POVRaySceneXMLPopUp();
00209       // QString GetPOVRaySceneXMLData();
00210       // void POVRayScenePreview();
00211 
00212    private:
00213 
00214       enum EExperimentState {
00215          EXPERIMENT_INITIALIZED = 0,
00216          EXPERIMENT_PLAYING,
00217          EXPERIMENT_FAST_FORWARDING,
00218          EXPERIMENT_PAUSED,
00219          EXPERIMENT_SUSPENDED,
00220          EXPERIMENT_DONE
00221       };
00222 
00223    private:
00224 
00225       CQTOpenGLWidget* m_pcOpenGLWidget;
00226       QString m_strIconDir;
00227       QString m_strTextureDir;
00228 
00229       EExperimentState m_eExperimentState;
00230 
00231       QAction* m_pcPlayAction;
00232       QAction* m_pcFastForwardAction;
00233       QAction* m_pcStepAction;
00234       QAction* m_pcResetAction;
00235       QAction* m_pcTerminateAction;
00236       QAction* m_pcPauseAction;
00237       QAction* m_pcCaptureAction;
00238       QAction* m_pcQuitAction;
00239       QSpinBox* m_pcDrawFrameEvery;
00240       QLCDNumber* m_pcCurrentStepLCD;
00241       QToolBar* m_pcExperimentToolBar;
00242       QMenu* m_pcExperimentMenu;
00243 
00244       QAction* m_pcShowCameraXMLAction;
00245       QList<QAction*> m_pcSwitchCameraActions;
00246       QActionGroup* m_pcSwitchCameraActionGroup;
00247       QDoubleSpinBox* m_pcFocalLength;
00248       QToolBar* m_pcCameraToolBar;
00249       QMenu* m_pcCameraMenu;
00250 
00251       QAction* m_pcPOVRayXMLAction;
00252       QAction* m_pcPOVRayPreviewAction;
00253       QMenu* m_pcPOVRayMenu;
00254 
00255       QAction* m_pcAboutQTAction;
00256       QMenu* m_pcHelpMenu;
00257 
00258       QStatusBar* m_pcStatusbar;
00259 
00260       QDockWidget* m_pcLogDock;
00261       QDockWidget* m_pcLogErrDock;
00262       QTextEdit* m_pcDockLogBuffer;
00263       QTextEdit* m_pcDockLogErrBuffer;
00264       CQTOpenGLLogStream* m_pcLogStream;
00265       CQTOpenGLLogStream* m_pcLogErrStream;
00266 
00267       bool m_bWasLogColored;
00268 
00269       CQTOpenGLUserFunctions* m_pcUserFunctions;
00270    };
00271 
00272 }
00273 
00274 #endif
```

---

Generated on 10 Jul 2018 for ARGoS by 
 1.6.1
